# Articles by Latin American Authors in Prestigious Journals Have Fewer Citations

**DOI:** 10.1371/journal.pone.0003804

**Published:** 2008-11-25

**Authors:** Rogerio Meneghini, Abel L. Packer, Lilian Nassi-Calò

**Affiliations:** 1 BIREME-PAHO–WHO, Latin-American and Caribbean Center on Health Sciences Information, São Paulo, Brazil; 2 DIS-Departamento de Informática Médica, Universidade Federal de São Paulo, São Paulo, Brazil; National Institute for Communicable Diseases, South Africa

## Abstract

**Background:**

The journal Impact factor (IF) is generally accepted to be a good measurement of the relevance/quality of articles that a journal publishes. In spite of an, apparently, homogenous peer-review process for a given journal, we hypothesize that the country affiliation of authors from developing Latin American (LA) countries affects the IF of a journal detrimentally.

**Methodology/Principal Findings:**

Seven prestigious international journals, one multidisciplinary journal and six serving specific branches of science, were examined in terms of their IF in the Web of Science. Two subsets of each journal were then selected to evaluate the influence of author's affiliation on the IF. They comprised contributions *(i)* with authorship from four Latin American (LA) countries (Argentina, Brazil, Chile and Mexico) and *(ii)* with authorship from five developed countries (England, France, Germany, Japan and USA). Both subsets were further subdivided into two groups: articles with authorship from one country only and collaborative articles with authorship from other countries. Articles from the five developed countries had IF close to the overall IF of the journals and the influence of collaboration on this value was minor. In the case of LA articles the effect of collaboration (virtually all with developed countries) was significant. The IFs for non-collaborative articles averaged 66% of the overall IF of the journals whereas the articles in collaboration raised the IFs to values close to the overall IF.

**Conclusion/Significance:**

The study shows a significantly lower IF in the group of the subsets of non-collaborative LA articles and thus that country affiliation of authors from non-developed LA countries does affect the IF of a journal detrimentally. There are no data to indicate whether the lower IFs of LA articles were due to their inherent inferior quality/relevance or psycho-social trend towards under-citation of articles from these countries. However, further study is required since there are foreseeable consequences of this trend as it may stimulate strategies by editors to turn down articles that tend to be under-cited.

## Introduction

Scientists from developing countries seek arduously to publish their papers in prestigious mainstream international journals. Submission acceptance influences their career advancement and success in obtaining research grant funding. In particular, many Latin American research funding agencies and institutional committees responsible for deciding on promotions or selection of candidates to academic positions, frequently base their decisions on the impact factor (IF, produced by the Journal Citation Report, JCR, Thomson-Reuters) of the journals where the articles of the applicant have been published. More developed countries have also followed this procedure, like Italy [1], Nordic countries [2], Canada [3] and Hungary [4] among others. The IF of a journal indicates the average number of citations the articles of this journal received among all journals indexed in this database in a given period of time. For instance, the two-year based IF for a journal in the year 2006 is obtained by dividing the number of citations received in 2006 for articles published in 2004 and 2005 by the number of these articles published in 2004 and 2005. The IF is accepted as a reasonable measurement of the quality of a journal but IFs can only be compared if potential bias is taken into consideration e.g. the journals being compared must belong to the same area of investigation [5]. The use of the average IF of the journals in which an author is published as a direct measurement of his/her quality is, however dangerously misleading. Seglen, for instance [2], draws attention to the fact that the most cited half of journal articles are cited 10 times more often than the less cited half. Therefore, the possibility is not negligible that two scientists with the same pattern of publications in journals (and therefore with a similar weighted average IF) may have very distinct rates of citations per article. Distinct citation trends in sub-areas covered by a journal further prejudice the comparison of scientists in the same area [6].

When dealing with the collective group data, however, the use of citation analysis agrees significantly with peer opinions. This has been the case for assessment of research departments [7] and of national PhD programs [8]. Also, many studies refer to a good fit between the opinion of peers on the quality of the articles in a journal and its IF (e.g. chapter 5, reference 5).

The question of the national contribution to the impact of an international journal has been pointed out [9]. As far as we know, however, there have been no studies on the trend of impact of articles published in a given journal with respect to author's affiliation. We hypothesize that affiliation affects IF of entire subsets of articles when compared with the IF of all articles published by the journal.

## Materials and Methods

We have chosen to analyze the output of the four LA countries with the highest number of publications in the Web of Science (WoS) data base: Argentina, Brazil, Chile and Mexico. In 2004 and 2005 the total contribution of these 4 countries represented 93.2% of the total LA WoS entries. This output was measured in six journals with a high reputation in their areas of knowledge and one multidisciplinary journal: Astrophysical Journal, Chemistry of Materials, Journal of the American Chemical Society, Journal of Biological Chemistry, Journal of Immunology, Physical Review Letters and Proceedings of the National Academy of Sciences of the USA. A total of 1244 articles published in the years 2004 and 2005 with the above country's authorships were found in the WoS database. These 1244 articles were divided in two subsets for each journal: one with LA authorship only (total of 219 articles) and another one including both non-collaborative and collaborative articles with other countries, virtually all being developed countries (219+1025 = 1244). The 2006 IFs of the two groups for each journal were calculated by the sum of citations given in 2006 to articles published in 2004 and 2005 divided by the number of articles published in these years. The same procedure was followed to calculate the IFs of groups of articles in these journals from five developed countries, namely, England, France, Germany, Japan and USA, for the purpose of comparison.

Attention must be given to the fact that the IF calculated according to this methodology does not correspond to the IF presented by the JCR, since the JCR and the WoS operate different journal collections. The reason for our using the WoS database is the availability of data to calculate the journal IF for countries which was not available in the standard JCR. This article and others present pitfalls in the standard calculation of IF [10] however, for the seven journals examined the overall WoS IF was 87.4±4.1% of the JCR IF, which would indicate that in the context of the present study the same conclusions would be achieved using the JCR IF.

## Results

The trend of lower IF for LA countries' articles is readily noticed in Table 1. For 22 out of 26 journal/“total country” subsets the IF was inferior to the total IF of the journal. One should notice that on average, 77% of the total subsets represented international collaborations with a developed country. When only the non-collaborative articles are considered all of the measurable IFs are considerably lower. To provide a broad picture for comparison, the average for the overall IF of the seven journals was 5.93; for the total (collaborative and non-collaborative) LA articles it was 5.04 and for the non-collaborative LA articles it was 3.38.

**Table 1:** The 2006 impact factor of Web of Science specialized Journals and subsets of articles from Argentina, Brazil, Chile and Mexico.

Journal		Total	Argentina		Brazil		Chile		Mexico	
			total	Country only	total	Country only	total	Country only	total	Country only
Astrophys. J.	IF 2006	**5.36**	**4.59**	**3.00**	**5.33**	**2.94**	**6.30**	**2.50**	**5.04**	**2.35**
	citations in 2006	27643	133	12	368	50	1027	20	887	47
	articles 2004+2005	5161	29	4	69	17	163	8	176	20
Chem. Materials	IF 2006	**4.60**	**1.80**	**2.33**	**1.68**	**1.40**	**–**	**–**	**3.59**	**2.00**
	citations in 2006	8276	9	7	32	14	0	0	43	6
	articles 2004+2005	1790	5	3	19	10	0	0	12	3
J. Am. Chem. Soc.	IF 2006	**6.55**	**4.71**	**3.25**	**9.25**	**5.67**	**–**	**–**	**6.41**	**4.00**
	citations in 2006	43558	66	13	148	34	0	0	205	12
	articles 2004+2005	6652	14	4	16	6	0	0	32	3
J. Biol. Chem.	IF 2006	**5.31**	**4.45**	**3.27**	**4.26**	**3.24**	**4.58**	**3.75**	**3.48**	**2.50**
	citations in 2006	63051	169	49	247	68	110	30	167	15
	articles 2004+2005	11875	38	15	58	21	24	8	48	6
Proc. Natl. Acad Sci. USA	IF 2006	**8.37**	**4.55**	**–**	**8.17**	**6.50**	**5.54**	**7.17**	**7.98**	**–**
	citations in 2006	56903	100	12	237	13	72	43	423	6
	articles 2004+2005	6798	22	1	29	2	13	6	53	1
J. Immunol.	IF 2006	**5.71**	**3.85**	**4.50**	**4.58**	**3.18**	**4.75**	**–**	**5.00**	**–**
	citations in 2006	22026	50	18	165	35	19	28	70	0
	articles 2004+2005	3860	13	4	36	11	4	1	14	0
Phys. Rev. Lett.	IF 2006	**5.63**	**4.73**	**2.45**	**6.78**	**3.39**	**3.15**	**2.40**	**6.80**	**2.62**
	citations in 2006	43796	336	27	983	122	41	12	877	34
	articles 2004+2005	7784	71	11	145	36	13	5	128	13
	**Total**	**5.93**	**4.10**	**3.13**	**5.72**	**3.76**	**4.87**	**3.95**	**5.47**	**2.69**

Data were collected from Thomson Reuters WoS data base. Two columns of IF are shown for each country for selected journals. One is for the total of articles of the country and the other for articles with affiliation of the country only, without collaboration. For each journal the corresponding 2006 citations of 2004+2005 articles and the number of 2004+2005 articles are shown, below each IF value .The dashed lines correspond to indefinite or imprecise IF for 0 articles or very low number of articles (typically 1).

If we consider the collaborative LA articles only, the average IF raises to 5.25 (not shown in Table 1) approaching the average of the overall IFs of the seven journals (5.93).

Table 2 has an equivalent framework to Table 1, except that it refers to five developed countries: England, France, Germany, Japan, and USA. It permits an important comparison with the LA countries. In these cases the differences in IF for the non-collaborative articles of countries and the overall IF of the journals is not significant: the average for the overall IF of the seven journals was 5.93; for the total (collaborative and non-collaborative) articles from these five countries it was 6.36 and for the non-collaborative articles it was 5.73.

**Table 2:** The 2006 impact factor of Web of Science specialized Journals and subsets of articles from England, France, Germany, Japan and USA.

Journal		Total	England		France		Germany		Japan		USA	
			total	Country only	total	Country only	total	Country only	total	Country only	total	Country only
Astrophys. J.	IF 2006	**5.36**	**7.51**	**3.36**	**6.35**	**4.00**	**8.38**	**4.60**	**5.80**	**3.78**	**5.80**	**5.16**
	citations in 2006	27843	3933	141	2101	96	4935	262	2835	775	22921	10793
	articles 2004+2005	5161	524	42	331	24	589	57	489	205	3953	2093
Chem. Materials	IF 2006	**4.60**	**4.68**	**5.25**	**3.33**	**3.61**	**5.20**	**5.12**	**4.49**	**4.26**	**5.05**	**5.31**
	citations in 2006	8276	426	273	642	408	874	502	924	648	3132	2560
	articles 2004+2005	1709	91	52	193	113	168	98	206	152	620	482
J. Am. Chem. Soc.	IF 2006	**6.55**	**6.91**	**7.07**	**6.08**	**5.82**	**6.86**	**6.76**	**6.57**	**6.94**	**7.14**	**7.16**
	citations in 2006	43586	2570	1527	1842	809	3444	1988	5581	5033	26443	22473
	articles 2004+2005	6852	372	216	303	139	502	294	850	725	3702	3137
J. Biol. Chem.	IF 2006	**5.31**	**5.68**	**5.69**	**5.12**	**4.85**	**5.57**	**5.35**	**5.48**	**5.00**	**5.72**	**5.65**
	citations in 2006	83051	4671	2151	4081	1953	5546	2700	7116	4269	38445	29022
	articles 2004+2005	11875	823	378	797	403	995	505	1299	854	6716	5138
Proc. Natl. Acad Sci. USA	IF 2006	**8.37**	**8.99**	**8.85**	**8.69**	**7.90**	**9.24**	**9.17**	**8.45**	**7.17**	**8.86**	**8.60**
	citations in 2006	56903	4369	16.46	3197	1146	4585	1733	3792	1556	43964	32418
	articles 2004+2005	6798	486	186	368	145	496	189	449	217	4964	3769
J. Immunol.	IF 2006	**5.71**	**6.15**	**5.98**	**5.57**	**5.17**	**6.13**	**5.88**	**6.52**	**5.23**	**6.01**	**5.70**
	citations in 2006	22028	1882	801	1426	672	2169	965	2328	1066	14241	10010
	articles 2004+2005	3860	306	134	256	130	354	164	357	204	2369	1756
Phys. Rev. Lett.	IF 2006	**5.63**	**5.95**	**4.94**	**5.81**	**4.68**	**6.08**	**5.53**	**6.30**	**5.00**	**6.27**	**6.05**
	citations in 2006	43796	4357	1018	6276	1742	9798	3479	5327	1961	22372	12264
	articles 2004+2005	7784	732	206	1080	372	1612	629	845	392	3568	2028
	**Total**	**5.93**	**6.55**	**5.88**	**5.85**	**5.15**	**6.78**	**6.06**	**6.23**	**5.34**	**6.41**	**6.23**

Data were collected from Thomson Reuters WoS data base. Two columns of IF are shown for each country for selected journals. One is for the total of articles of the country and the other for articles with affiliation of the country only, without collaboration. For each journal the corresponding 2006 citations of 2004+2005 articles and the number of 2004+2005 articles are shown below each IF value.

## Discussion

The visibility of scientific research, as a rule, benefits from increasing collaboration [13]. In the present study international collaboration had a minor effect on the IF when referred to scientifically developed countries. The exception was the Astrophysical Journal for which the non-collaborative articles had an average IF of 4.14 and the total articles an average IF of 6.77. Probably this has to do with the need of great telescopes for obtaining the most impacting results, located in Canarias, Hawaii, South Africa, Texas and Chile and to a privileged access to the Hubble telescope. In this case the USA would be less affected in a non-collaborative work since six of the ten biggest telescopes are located in their territory and the Hubble telescope belongs to NASA-USA.

The situation is different, however, in LA countries. An interesting point is the extent of collaboration in articles from these seven journals: the percentage of 77% is considerably higher than those of the collaborative effort of these four LA countries in the context of the whole WoS database: Argentina 40.3%, Brazil 26.7%, Chile 50.2% and Mexico 53.9%. This could be a measure of the effort required to publish in these seven prestigious journals and the importance of international collaboration to accomplish it.

The most important question in regard to these data is why the subsets of the non-collaborative articles of LA countries present such a low IF as compared to the overall IF of the journals? In principle one would expect a relatively homogenous review process for all manuscripts submitted to a given journal and the same rigor for their acceptance. However, the 219 non-collaborative articles from LA fall dramatically behind the average impact of these seven journals. The groups of non-collaborative articles that were closest to the overall IF were Argentina /J. Immunol. (4.50/5.71), Brazil / J. Am. Chem. Soc. (5.67/6.55) and Chile / Proc. Natl. Acad. Sci. USA (7.17/8.37). Brazil has strong research groups in chemistry [11] and Argentina has a strong tradition in immunology with one Nobel Prize in the category ‘Physiology or Medicine’ [12]. This would seem to indicate that LA articles are ignored with the possible exception of those centers of excellence. Presently, it is impossible to make a judgment as to whether this is due to the quality/relevance of the these articles or if articles with LA authors, without international collaboration, are destined to be under-cited due to social-psychological reasons. Although not yet an object of analysis, it is noticeable that many Brazilian authors envisaging publications in mainstream journals tend to produce reference lists containing a majority of prominent authors and prestigious journals and avoid citations of their compatriots, as if this would give more weight to their publications [14], [15]. One may wonder if a similar behavior also occurs with authors from developed countries, leading to a significant under citation of LA articles.

Regardless of the reason for the under citation of non-collaborative LA articles a drawback may be foreseeable in regard to the competition for editorial space in the high-status journals: would an Editor, concerned about the journal IF, consider that the acceptance of a LA article might weight against its value? After all, it is known that several strategies for increasing the IF are used by editors [16]. Why not consider strategies for protection against a decrease of IF value?

In conclusion, scientometric data render it possible to detect the under-citation trend of non-collaborative LA articles of specific prestigious journals but provides no elements to decide the basis for this phenomenon. Possible reasons could include psycho-social bias or real differences in scientific relevance of these articles. The only way to address this argument would be to conduct a detailed peer analysis of the articles, trying to establish a correlation between citation and quality, as has been done in similar circumstances [7], [8].

## References

[1] Calza L, Garbisa S (1995). Italian professorships.. Nature.

[2] Seglen PO (1997). Why the impact factor of journals should not be used for evaluating research.. British Medical Journal.

[3] Taubes G (1993). Measure for measure in science.. Science.

[4] Vinkler P (1986). Evaluation of some methods for the relative assessment of scientific publications.. Scientometrics.

[5] Moed HF (2005). Citation analysis in research evaluation.

[6] Postma E (2007). Inflated impact factors? The true impact of evolutionary papers in non-evolutionary journals.. PLoS One.

[7] Norris M, Oppenheim C (2003). Citation counts and the research assessment exercise V.. J Documentation.

[8] Ostriker JP, Kuh CV (2003). Assessing Research-Doctorate Programs: A Methodology Study.

[9] Schubert A, Glanzel W, Braun T (1989). Scientometric data files. A comprehensive set of indicators on 2649 journals and 96 countries in all major fields and subfields 1981–1985.. Scientometrics.

[10] Moed HF, van Leeuwen THN, Reedijk J (1999). Towards appropriate indicators of journal impact.. Scientometrics.

[11] Vieira CL (1999). Chemistry: Brazil Lobbies for First Nobel.. Science.

[12] Karpas A (2002). César Milstein (1927–2002): a somewhat personal reflection.. Trends in Immunology.

[13] Glanzel W, Schubert A, Moed HF, Glanzel W, Schmoch U (2006). Analyzing scientific networks through co-authorship.. Handbook of quantitative science and technology research.

[14] Pinto AC, de Andrade JB (1999). Impact factor of scientific journals: what is the meaning of this parameter? Quím.. Nova.

[15] Meneghini R, Packer AL (2007). Is there science beyond English?. Embo reports.

[16] The PLoS Medicine Editors (2006). The Impact Factor Game.. PLoS Med.

